# The European Midwives Association call for action to protect our midwives in delivering best care amidst the COVID-19 pandemic

**DOI:** 10.18332/ejm/120443

**Published:** 2020-04-15

**Authors:** Joeri Vermeulen, Mervi Jokinen

**Affiliations:** 1 European Midwives Association, Antwerp, Belgium; 2 Department of Health Care, Knowledge Centre Brussels Integrated Care, Erasmus Brussels University of Applied Sciences and Arts, Brussels, Belgium; 3 Department of Public Health, Faculty of Medicine and Pharmacy, Biostatistics and Medical Informatics Research Group, Vrije Universiteit Brussel (VUB), Brussels, Belgium; 4 The Royal College of Midwives, London, United Kingdom

**Keywords:** COVID-19, European midwives, pregnancy, childbirth, midwifery organisation, midwives

It is now more than ever that midwifery organisations recognise their role in supporting midwives to work as safely as possible, while facilitating them to keep the focus on family-centred care. Since the COVID-19 outbreak we hear of midwifery organisations all over Europe uniting to lobby for adequate support in their quest to continue to provide safe quality care. As midwives we need to ensure we are engaged in developing appropriate guidelines and protocols during this challenging, constantly changing environment. In several European countries midwifery organisations made an urgent appeal to the policymakers for sufficient Personal Protective Equipment (PPE), so that midwives can continue their role safely.

Likewise, the European Midwives Association (EMA), jointly with eleven other European health professional organisations, have urged authorities to guarantee protection to those working on the front lines against COVID-19^1^. Staff must be provided with PPE and must be regularly tested; the increased number of infected health professionals is staggering and unfortunately some have succumbed to the viral condition. Health professionals are operating under very challenging conditions presented by the pandemic. Working in these conditions takes its toll both on the physical and mental health and appropriate support services must urgently be put in place. But remember, we all play an important role in supporting each other.

There is a lack of evidence about what is actually happening in maternity care in Europe^2^, but in most countries maternity care is under pressure since the outbreak. EMA has sent a short survey to all members asking some basic information to support or question the current anecdotal evidence we receive. This has been done with support of Royal College of Midwives UK who have already surveyed their own membership.

We hear new ways of providing antenatal care and antenatal group lessons are being explored and virtual technology is replacing face-to-face encounters. One of the biggest concerns for women discussed in social media is the banning of birth partners from being present at the birth. However, in most hospitals the partner is still allowed to be present at the birth. In some hospitals we hear that births are electively induced at 40 weeks, while skin-to-skin and breastfeeding might be unnecessarily discouraged as it is considered potentially unsafe. The continuity of care is essential now more than ever but safeguarding it is challenging. As in all pandemics, the recommendation is to provide as much care as possible in the community, therefore early transfer to home after birth is becoming more regular. However, some countries may not have the capacity for this in their current system configurations. Our role is to make sure that these women and babies continue to receive the care they need, reducing their anxiety and remembering that both their mental and physical health are paramount. In turn, we need to ensure the safety of midwives working in the community with provision of adequate PPE where necessary.

Even during a pandemic, we foster that public health concerns are balanced fairly against the need to respect autonomy, informed choice and evidence^3^. The International Confederation of Midwives^4^ expressed concerns that human rights might be violated by the introduction of inappropriate protocols for management of maternity care in response to the pandemic. These protocols are not based on current reputable evidence and are harmful to women and their babies.

There is no doubt that those working within maternity care are doing everything they can to best support mother and newborn, but maternity care professionals need to be supported too. Health professionals are every country’s most valuable resource and need adequate support to provide the best quality care they can to women and newborns in exceptionally trying circumstances^5^. Therefore, EMA urges national and EU authorities to guarantee protection and acceptable working conditions so that midwives can keep the focus on safe and respectful family-centred care, now more than ever.
